# A nonsense mutation in *CRYGC* associated with autosomal dominant congenital nuclear cataract in a Chinese family

**Published:** 2008-07-09

**Authors:** Ke Yao, Chongfei Jin, Ning Zhu, Wei Wang, Renyi Wu, Jin Jiang, Xingchao Shentu

**Affiliations:** Eye Center of the 2nd Affiliated Hospital, Medical College of Zhejiang University, Hangzhou, China

## Abstract

**Purpose:**

To identify the genetic defect associated with autosomal dominant congenital nuclear cataract in a Chinese family.

**Methods:**

Family history and phenotypic data were recorded, and the phenotypes were documented by slit lamp photography. The genomic DNA was extracted from peripheral blood leukocytes. All the exons and flanking intronic sequences of *CRYGC* and *CRYGD* were amplified by polymerase chain reaction (PCR) and screened for mutation by direct DNA sequencing. Structural models of the wild type and mutant γC-crystallin were generated and analyzed by SWISS-MODEL.

**Results:**

Sequencing of the coding regions of *CRYGC* and *CRYGD* showed the presence of a heterozygous C>A transversion at c.327 of the coding sequence in exon 3 of *CRYGC* (c.327C>A), which results in the substitution of a wild type cysteine to a nonsense codon (C109X). One and a half Greek key motifs at the COOH-terminus were found to be absent in the structural model of the mutant truncated γC-crystallin.

**Conclusions:**

A novel nonsense mutation in *CRYGC* was detected in a Chinese family with consistent autosomal dominant congenital nuclear cataract, providing clear evidence of a relationship between the genotype and the corresponding cataract phenotype.

## Introduction

Hereditary congenital cataract (OMIM 604307) is an opacification of the eye lens that frequently results in visual impairment or even blindness during infancy or early childhood. Despite the great advances in the clinical management of cataracts as well as a better understanding of lens structure and function, congenital cataract remains a leading cause of blindness in children worldwide [1,2]. Irreversible visual loss can result if prompt treatment is not performed on these patients. Congenital cataracts are considered to be both phenotypically and genetically heterogeneous [3-5]. The water-soluble lens crystallins account for nearly 90% of the total lens proteins and play essential roles in maintaining the lens transparency [6]. Therefore, crystallins are good candidate genes for congenital cataract.

Crystallins are subdivided into α-, β-, and γ-crystallins with the γ-crystallin gene cluster subdivided into six genes, *CRYGA*-*CRYGF*. Only *CRYGC* (OMIM 123680) and *CRYGD* (OMIM 123690) are known to encode abundant lens γ-crystallins in humans [7,8]. The γ-crystallins have two domains with each domain composed of two exceptionally stable protein structures called “Greek key” motifs [9]. The γ-crystallins are monomeric with a molecular mass of 21 kDa and comprise about 40% of the total proteins in the mouse lens and 25% in the human lens [6,10]. As reported, mutations in *CRYGC* and *CRYGD* have been identified to cause isolated autosomal dominant congenital cataracts [11,12] as a result of altered stability, association, and/or solubility of γ-crystallins [13-16]. Indeed, in our previous study, we reported heterozygous mutations in *CRYGD* in a four-generation Chinese family with distinct fasciculiform cataract [17].

In the present study, we investigated a large Chinese family with autosomal dominant congenital nuclear cataract and detected a novel chain-termination mutation in *CRYGC* that cosegregated with the disease in the family.

## Methods

### Patients and clinical data

**Figure 1:**
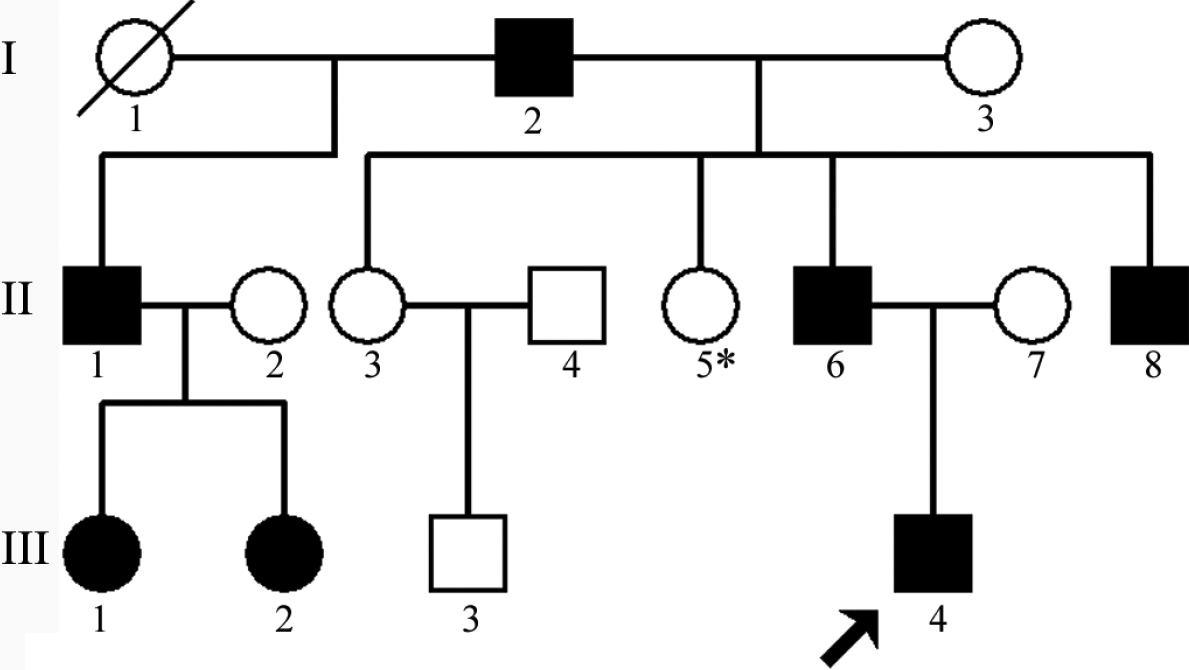
Pedigree of the autosomal dominant congenital cataract. The proband is marked with an arrow. Squares and circles indicate males and females, respectively. Black and white symbols denote affected and unaffected individuals, respectively. A slash through the symbol signifies that the family member is deceased. Thirteen individuals (seven affected and six unaffected) from the family were enrolled and underwent ophthalmologic examinations and genotyping in the study (II:5, marked by an asterisk, did not participate in the study).

A family of three generations was ascertained through the Eye Center of the 2nd Affiliated Hospital (Medical College of Zhejiang University, Hangzhou, China). Appropriate informed consent from each participant was obtained in accordance with the Zhejiang Institutional Review Board, and the study protocol adhered to the guidelines of the Declaration of Helsinki. Thirteen individuals (seven affected and six unaffected) from the family were enrolled in the study (Figure 1). Affected status was determined by a history of cataract extraction or ophthalmologic examination on presentation including visual function, slit lamp examination, and fundus examination with the dilated pupil. The phenotype was documented by slit lamp photography. Fifty subjects without diagnostic features of congenital cataract were recruited from the Chinese Han population in our medical examination center to serve as normal controls.

### Genomic DNA preparation and molecular analysis

Blood specimens (5 ml) from all the patients and available family members were collected in EDTA. Genomic DNA was isolated as previously described [18]. Since the number of mutations leading to dominant cataracts was fairly high in the human *CRYG* gene cluster, *CRYGC* and *CRYGD* were taken as a priority to be screened as the candidate genes. The exons and flanking regions of *CRYGC* and *CRYGD* in patients II:6 and III:4 were amplified and sequenced using the primers listed in (Table 1). The cycling conditions for PCR were 38 cycles of 95 °C for 25 s, 55 °C for 25 s and 72 °C for 35 s, preceded by 5 min at 95 °C and followed by a final elongation step at 72 °C for 10 min. Any interesting sequence variation of a mutation suspect was later confirmed in the rest of the patients and unaffected family members by bidirectional sequencing of the particular exon.

**Table 1:** Polymerase chain reaction primers and product sizes.

**Name**	**Primer sequence (5′-3′)**	**Product size (bp)**
GC1,2F	5′ TGCATAAAATCCCCTTACCGCTGA 3′	522
GC1,2R	5′ ACTCTGGCGGCATGATGGAAATC 3′	
GC3F	5′ AGACTCATTTGCTTTTTTCCATCCTTCTTTC 3′	407
GC3R	5′ GAAAGAATGACAGAAGTCAGCAATTGCC 3′	
GD1,2F	5′ CTTATGTGGGGAGCAAACT 3′	619
GD1,2R	5′ CAGCAGCCCTCCTGCTAT 3′	
GD3F	5′ TGCTTTTCTTCTCTTTTTATTTCTGGGTCC 3′	400
GD3R	5′ AGTAAAGAAAGACACAAGCAAATCAGTGCC 3′	

### Comparative modeling of γC-crystallins

Three-dimensional structures of the wild type and the mutant γC-crystallin were modeled on the basis of the crystal structure of the mouse γC-crystallin chain A [19]. The homology models were generated by SWISS-MODEL and analyzed in the Swiss-PdbViewer, version 3.7 (GlaxoSmithKline R&D, UK) [20-22].

## Results

### Clinical evaluation

**Figure 2:**
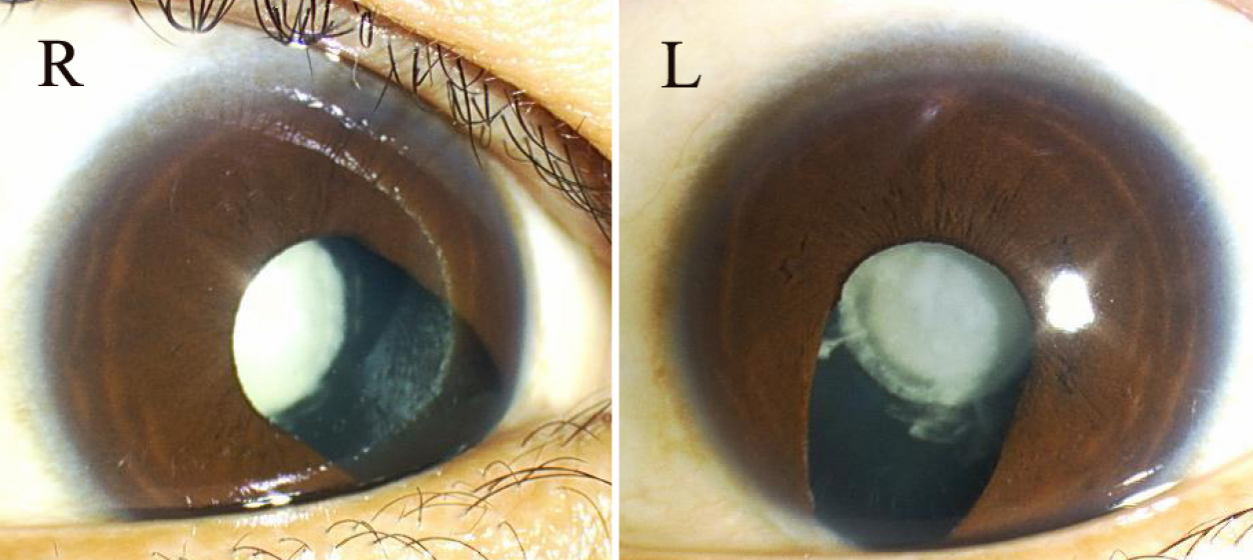
Slit lamp photographs of affected individual II:6. Lens opacities were located in the embryonal, fetal, and infantile nuclei of the lens while the cortex remained transparent. The patient underwent iridectomy on both eyes in his early childhood.

We identified isolated autosomal dominant congenital nuclear cataract in a three-generation Chinese family. Opacification of the lens was bilateral and consistent in all of the affected individuals. All embryonal, fetal, and infantile nuclei of the lens were opacified while the cortex remained transparent (Figure 2). Visual acuity ranged from light perception to 0.15 in the unoperated eyes and from 0.20 to 0.02 in the eyes that had undergone iridectomy during childhood. Obvious nystagmus was observed in all the patients except the 10-month-old proband who received phacoemulsification surgery in both eyes on presentation. There was no history of other ocular or related systemic abnormalities in the family aside from age-related changes.

### Mutation analysis

**Figure 3:**
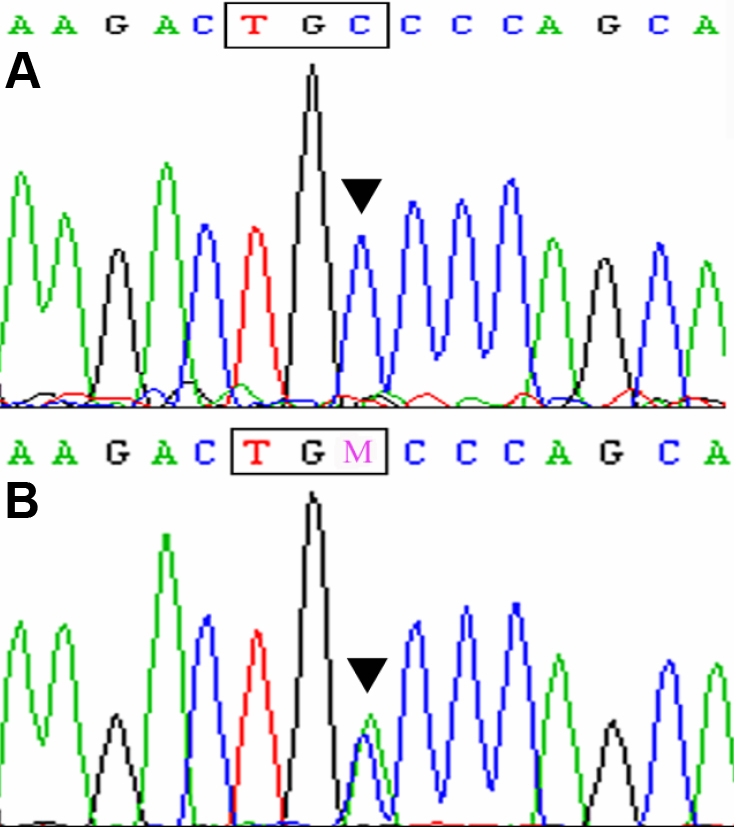
Forward sequence analysis of *CRYGC*. **A**: The sequence of an unaffected member (individual II:7) is shown. **B**: The sequence of an affected member (individual II:6) is shown. A heterozygous mutation was detected in the exon 3 of *CRYGC* (c.327C>A).

Direct sequencing was performed to cover exons and flanking intron-exon boundary sequences. A heterozygous C>A transversion was identified at c.327 in exon 3 of *CRYGC* in all the affected members but not in any of the unaffected family members (Figure 3). This mutation resulted in the substitution of a wild type cysteine to a nonsense codon (C109X). The variant was completely absent in 100 chromosomes of 50 unrelated controls.

### Comparison of wild type and mutant γC-crystallin structures

**Figure 4:**
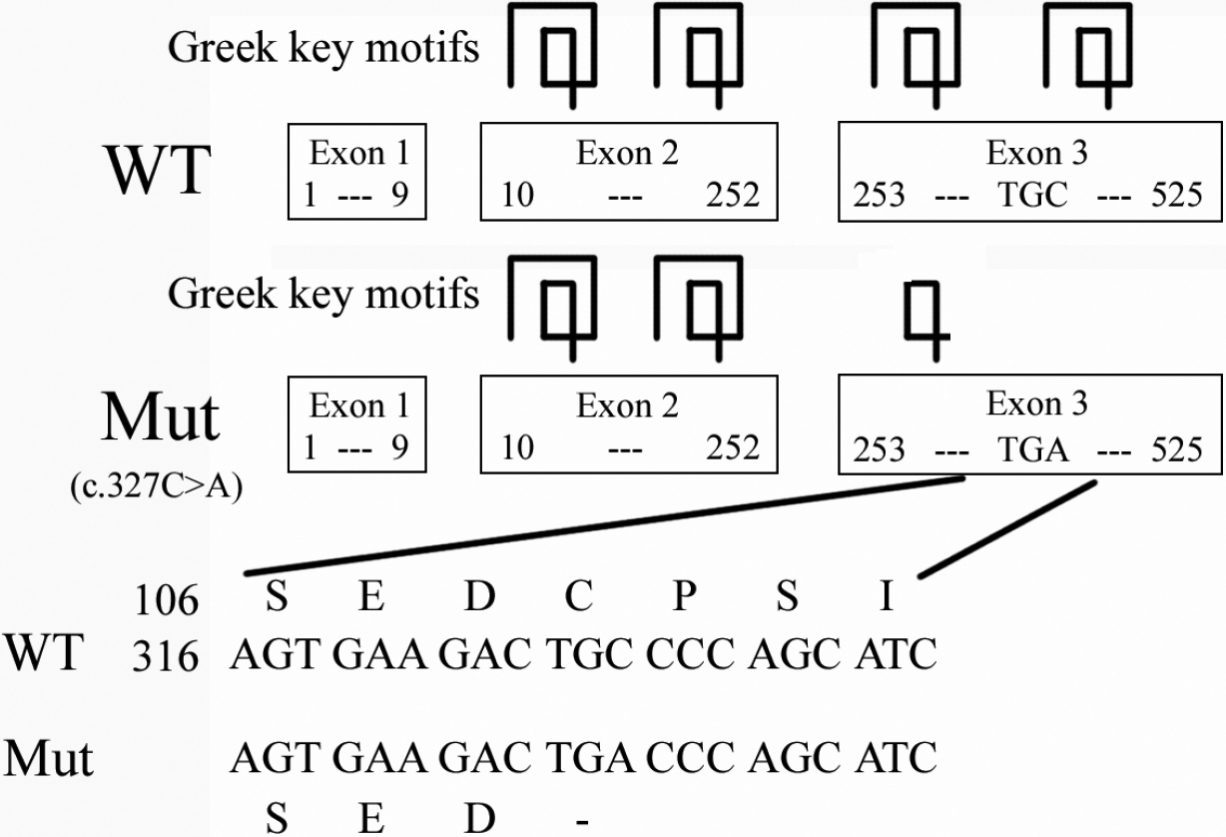
Influence of the mutation (c.327C>A) on γC-crystallin translation. The C>A substitution at c.327 in exon 3 leads to a premature stop codon at codon 109. A truncated protein (108 amino acids) is putatively generated in addition to a wild type γC-crystallin (174 amino acids).

**Figure 5:**
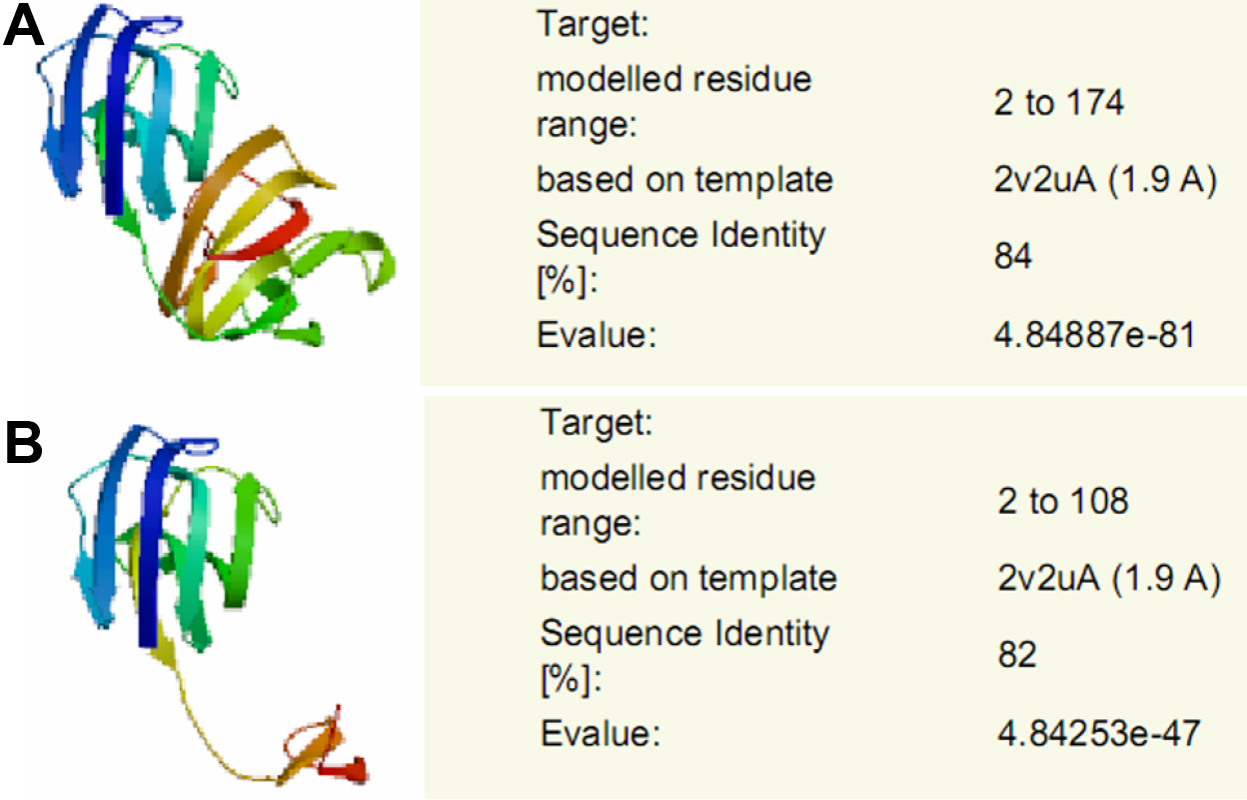
Structural modeling of the wild type and mutant γC-crystallins. The structure modeling is based on the X-ray determined coordinates of mouse γC-crystallin chain A using SWISS-MODEL. **A**: A structural model of the wild type γC-crystallin with 84% sequence identity is demonstrated. **B**: A structural alteration of the mutant γC-crystallin with 82% sequence identity is shown. Highly symmetric structure of γC-crystallin is disrupted when 66 amino acids are truncated from the COOH-terminus of γC-crystallin as result of c.327C>A mutation.

The C>A transversion at position c.327 in exon 3 led to a premature stop codon at codon 109. A truncated protein with 108 amino acids was putatively generated, 66 amino acids less than the wild type γC-crystallin, which possesses 174 amino acids (Figure 4). When modeled by SWISS-MODEL, one and a half Greek key motifs at the COOH-terminus were found to be absent in the three-dimensional structural model of the mutant γC-crystallin (Figure 5).

## Discussion

In the present study, we detected a novel mutation (c.327C>A) in exon 3 of *CRYGC* in a Chinese family with autosomal dominant congenital nuclear cataract. The cataract phenotype was consistent among all the affected family members, providing a clear relationship between the genotype and the corresponding cataract phenotype. The opacification in the nuclei but not in the cortex could be explained by the fact that monomeric γC-crystallin, the major type of γ-crystallin expressed in the young human lens, is synthesized in the early life span and localized only in the central regions of the mature/aging eye lens [23,24].

**Figure 6:**
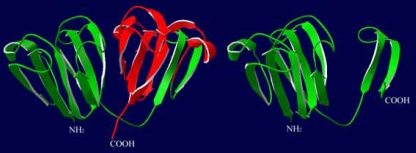
Comparative modeling of the full length and truncated γC-crystallins. The structural modeling was analyzed in Swiss-PdbViewer (version 3.7). When comparing the full length (left) and truncated γC-crystallins (right), the six COOH-terminal β-strands are truncated in the mutated γC-crystallin (the strands are shown in red).

To our knowledge, four mutations in *CRYGC* have been reported in the literature (listed in Table 2) [11,25-27]. The mutation detected in our present study, c.327C>A, creates a premature stop codon (C109X) and results in an in-frame stop codon at nucleotide 75 of exon 3 that may cause a truncation of 66 amino acids from the COOH-terminus of γC-crystallin. The secondary structure predicted by the Protein Prediction program (PHD) [28] shows that there are 16 β-strands (β1-β16) in γC-crystallin. The Cys109 residue located between the β10-strand and β11-strand is replaced by a nonsense codon, resulting in the loss of six β-strands after the β10-strand (Figure 4). Consequently the highly symmetric structure of γC-crystallin is lost (Figure 6).

**Table 2:** Human *CRYGC* mutations associated with congenital cataract.

**Cataract Phenotypes**	**Mutations**	**Exons**	**Effects**	**Protein domains**	**References**
Coppock-like	c.13A>C	1	p.Thr5Pro	GKM 1	[11]
Zonular pulverulent	c.123insGCGGC	2	p.Gly41delinsGlyfsX62	C-td 3 GKM loss	[25]
Lamellar/nuclear	c.502C>T	3	p.Arg168Trp	GKM 4	[26,27]
Nuclear	c.327C>A	3	p.Cys109X	C-td 1.5 GKM loss	this study

GKM=Greek key motif; C-td=COOH terminal domain.

Thus far, wild type human γC-crystallin has not been crystallized. Therefore, homology models for wild type and mutant human γC-crystallin are usually built based on the X-ray determined coordinates of mouse γC-crystallin chain A. The C109X mutation interferes with the formation of two COOH-terminal Greek key motifs. Although the function of the Greek key motifs has not been elaborated in detail, computer-based analysis suggests that it may be responsible for particular protein–protein interactions in the lens, and it is postulated to be critical in the maintenance of lens transparency [29].

It is reported that self-aggregation or quaternary structural alteration of γ-crystallin is responsible for the phenotypic association with lens opacification as well as cataractogenesis [30,31]. The truncated γC-crystallin may change the folding properties of γC-crystallin as it has been shown in a previous investigation that the COOH-terminal domain folds before and nucleates the folding of the NH_2_-terminal domain in human γD-crystallin refolding [32]. The relatively loose or partially unfolded structure of mutant γC-crystallin may be susceptible to aggregation and insolubilization, which leads to cataract formation [13]. Another possible consequence of the C109X mutation may be related to the disturbances of the interactions between γC-crystallin and other crystallins [16,33]. The truncated γC-crystallin in the present study may cause a decrease or even complete loss of the ability to interact with other crystallins and may result in congenital cataract.

In conclusion, the novel nonsense mutation (c.327C>A) in *CRYGC* in this Chinese family is associated with isolated autosomal dominant congenital nuclear cataract, giving evidence of a clear relationship between the genotype and the corresponding cataract phenotype. The possible influence of the mutation on the structure as well as the function of γC-crystallin will require further investigation.
